# 

**Published:** 1906-09-01

**Authors:** 


					.   Sept. Ibt, 1906.
) TSE HOSPITAL.
PITAL
JT -   I ZHN INFIRMARY
Vol. XL. [a Newspaper!] SATURDAY,
Sept. 1st, 1906.
-nuaniiLAAHA-nua nisu ausaou^ x-
[S?n"'STem'] PRICE THREEPENCE

				

